# Survey of Topical Steroid Usage Patterns After Descemet Membrane Endothelial Keratoplasty

**DOI:** 10.1097/ICO.0000000000003953

**Published:** 2025-08-05

**Authors:** Matilde Buzzi, Alexander M. Collins, Rhea Suribhatla, Sarah Schimansky, Saumya Yadav, Kieren Darcy, Derek Tole, Omar Elhaddad

**Affiliations:** * Bristol Eye Hospital, University Hospitals Bristol and Weston NHS Foundation Trust, Bristol, United Kingdom;; † Bristol Medical School, University of Bristol, Bristol, United Kingdom;; ‡ Bristol Royal Infirmary, University Hospitals Bristol and Weston NHS Foundation Trust, Bristol, United Kingdom; and; § Faculty of Medicine, Alexandria University, Egypt.

**Keywords:** corticosteroids, Descemet membrane endothelial keratoplasty, Fuchs endothelial corneal dystrophy, pseudophakic bullous keratopathy, graft rejection

## Abstract

**Purpose:**

To investigate global practice patterns of topical steroid treatment after Descemet Membrane Endothelial Keratoplasty (DMEK).

**Methods:**

An anonymized electronic questionnaire was disseminated to KeraNet members in March 2024, exploring postoperative corticosteroid use in three scenarios: 1) post-DMEK for Fuchs endothelial corneal dystrophy (FECD), 2) post-DMEK for pseudophakic bullous keratopathy (PBK), and 3) postrepeat DMEK after previous graft failure.

**Results:**

Seventy-eight responses were obtained from ophthalmologists in 15 countries. Participants performed a median of 37.5 DMEKs per year (mean 45.8; SD 46.7; range 2–300). Prednisolone 1% was the preferred topical steroid during the 12 months post-DMEK for FECD, in the 24 months post-DMEK for PBK, and after 24 months postrepeat grafts. Thirty-four surgeons (44%) discontinued topical steroids at 24 months post-DMEK for FECD, whereas 41 (53%) continued steroid drops indefinitely. For PBK, 36% of surgeons ceased topical steroid use at 24 months; 54% continued indefinitely. In repeat DMEKs after previous graft failure, 63% reported indefinite topical steroid treatment. Variability was also noted in long-term follow-up regimens: 45% of surgeons discharge their patients between postoperative years 1 to 5. The remaining 55% of respondents opted for indefinite patient follow-up.

**Conclusions:**

We identified significant variation in postoperative corticosteroid therapy after DMEK for FECD, PBK, and postgraft failure. This heterogeneity warrants further investigations exploring rates of graft rejection and failure across different postoperative steroid regimens. An international consensus would aid in standardizing postoperative care.

## Abstract 2

Supplemental Digital Content is Available in the Text.

Endothelial keratoplasty (EK) has led to significant advances in corneal transplantation. It is now the procedure of choice for endothelial dysfunction, for example Fuchs endothelial corneal dystrophy (FECD) and pseudophakic bullous keratopathy (PBK). Compared with Descemet stripping automated endothelial keratoplasty (DSAEK), Descemet membrane endothelial keratoplasty (DMEK) allows shorter postoperative recovery time, smaller refractive error change, lower rejection rates, and better cost-effectiveness in quality-adjusted life years.^1–3^ Subsequently, DMEK has gained widespread adoption, with procedure numbers steadily rising over the past decade.^4^

Topical steroids are the mainstay in ocular immunomodulatory and anti-inflammatory therapy after corneal transplantation. Their use in minimizing risk of graft rejection and failure must be considered alongside their potential to induce adverse effects, such as ocular hypertension and glaucoma.^5,6^ Steroid regimens for the prevention of allograft rejection after EK vary considerably by topical steroid type and concentration, frequency, and duration of treatment reported.^7^ There is ongoing debate regarding the lifelong use of topical steroids after EK.^7–9^

Previous surveys investigating steroid usage patterns after EK, published in 2011 and 2021, identified topical prednisolone 1% as corneal surgeons' preferred agent, and a tendency to taper steroid drop frequency incrementally over the first postoperative year.^10,11^ However, an updated assessment of ophthalmologists’ postoperative management pattern is warranted to inform best practice, particularly given the growing popularity of DMEK surgery, availability of longer follow-up data on graft rejection, and introduction of new medications.

The purpose of this study is to investigate the current practice patterns among corneal surgeons internationally regarding postoperative topical steroid treatment after DMEK or combined cataract surgery and DMEK (phaco-DMEK) in 3 different clinical scenarios (FECD, PBK, and repeated graft after failed DMEK), and to compare our findings with practice patterns reported in previous surveys.

## MATERIALS AND METHODS

This cross-sectional study employed a nonrandom sampling method to obtain responses from ophthalmologist subscribers to Kera-Net, the official online mailing list of the Cornea Society. A nine-item electronic questionnaire was hosted on Qualtrics (Provo, UT), a General Data Protection Regulation-compliant survey platform accessible via desktop and mobile devices. All responses to the survey were anonymous. Dissemination of the survey took place in March 2024. Each Kera-Net member was sent 2 invitations to participate in the study.

The 9-question survey (see Appendix, Supplemental Digital Content 1, http://links.lww.com/ICO/B871) began with one demographic question and one question about DMEK surgical volumes. The remaining seven questions explored practice patterns on postoperative topical steroid use in the context of routine DMEK with or without cataract surgery for FECD and PBK, and as part of management of previous graft failure. All questions in the survey allowed for free-text responses.

Descriptive analysis was conducted using Apple Numbers (Apple Inc., CA), and figures were generated using the same software. Any incomplete responses were removed before data analysis to ensure integrity of the study.

## RESULTS

**FIGURE 1.**
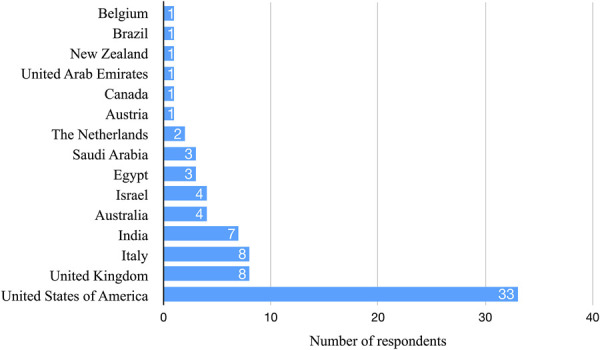
Country of practice of surveyed corneal surgeons (n = 78).

A total of 78 survey responses were obtained from participants in 15 different countries around the world. The United States (42.3%, n = 33), the UK (10.3%, n = 8), and Italy (10.3%, n = 8) were the 3 most common countries of practice among our respondents, with the full breakdown shown in Figure 1. Participants performed a median of 37.5 DMEKs per year (mean 45.8; SD 46.7; range 2–300).

### Topical Steroids After Routine DMEK or Phaco-DMEK for FECD

**FIGURE 2.**
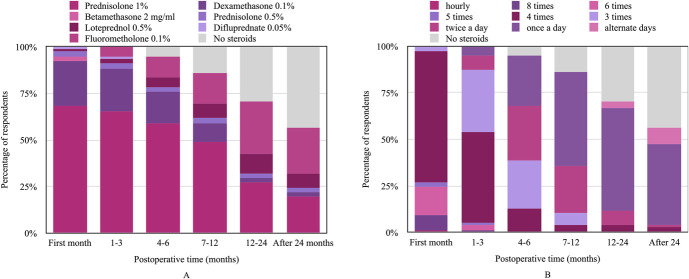
Distribution of surveyed corneal surgeons' (n = 78) (A) preferred topical steroid; (B) drop administration frequency for postoperative management after a routine DMEK or phaco-DMEK for FECD.

During the first month after routine or phaco-DMEK surgery for FECD, most of the surveyed surgeons prescribed prednisolone 1% eye drops (n = 53, 68%), followed by dexamethasone 0.1% (n = 19, 24%). Alternative topical steroids were prescribed less commonly (Fig. 2A). Regarding frequency of drop administration, most surgeons reported prescribing steroids 4 times a day (QID) during the first postoperative month (n = 55, 71%), as shown in Figure 2B.

During postoperative months 2 to 12, most participants described continuing prednisolone 1% eye drops (n = 51, 65% during months 1–3; n = 46, 59% during months 4–6; n = 38, 49% during months 7–12), with an increasing percentage of lower potency steroid prescriptions [fluorometholone 0.1% (FML) and loteprednol 0.5%]. After 12 months postoperatively, most surgeons reported switching to FML once a day (OD) (n = 22, 28% during months 12–24; n = 19, 24% after 24 months) with prednisolone 1% being the second most commonly prescribed eye drop (n = 21, 27% during months 12–24; n = 15, 19% after 24 months). The remaining surgeons continued to prescribe loteprednol (n = 8, 10% during months 12–24; n = 6, 8% after 24 months), dexamethasone 0.1% (n = 2, 3% during months 12–24 and after 24 months), or prednisolone 0.5% (n = 2, 3% during months 12–24 and after 24 months) throughout years 2 and 3. Twenty-three surgeons (29%) reported stopping steroid use after 12 months and 34 (44%) after 24 months.

Surgeons reported an incremental reduction in steroid eye drop frequency over time (Fig. 2B). Four clinicians reported stopping the topical steroids after 3 months post-DMEK surgery, compared with 23 (29%) at 12 months. After 24 months post-DMEK surgery, 34 surgeons (44% of the total) had stopped steroid prescription. Conversely, 41 (53%) clinicians opted to maintain treatment indefinitely, conditional on good control of intraocular pressure (31 respondents with OD administration regime, seven respondents on alternate days, two respondents four times a day, and one respondent twice a day). Moreover, two respondents continue treatment OD for 5 years; 1 respondent continues OD through year 3, followed by alternating days through year 4, and twice weekly through year 5.

### Topical Steroids After Routine DMEK for PBK

**FIGURE 3.**
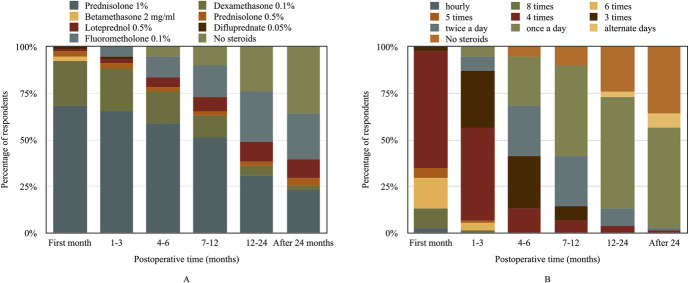
Distribution of surveyed corneal surgeons' (n = 78) (A) preferred topical steroid; (B) drop administration frequency for postoperative management after a routine DMEK for PBK.

For the first 12 months after routine DMEK for PBK, all participants reported prescribing the same steroid formulation as for their patients with FECD (Fig. 3A), but with increased frequency and duration of administration (see Fig. 3B). At 12 to 24 months postoperatively, most of the surgeons continued to prescribe prednisolone 1% eye drops (n = 24, 31% during months 12–24; n = 18, 23% after 24 months), with FML being the second most commonly prescribed agent. After 24 months, a total of 28 (36%) participants had ceased steroid eye drop use, whereas 50 (64%) respondents continued topical steroids (42 respondents OD, six respondents on alternate days, one respondent twice a day, and one respondent four times a day). Most surgeons continue indefinitely, whereas three surgeons for 5 years, two surgeons for 3 years, and one surgeon OD through year 3, then on alternate days through year 4, and twice weekly during year 5.

### Topical Steroids After DMEK for Graft Failure (Repeated Graft)

**FIGURE 4.**
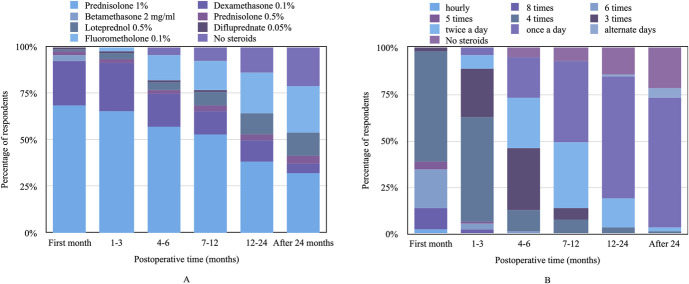
Distribution of surveyed corneal surgeons' (n = 78) (A) preferred topical steroid; (B) drop administration frequency for postoperative management after DMEK after previous failed DMEK (repeated graft).

For the first month after DMEK for a failed graft, all participants reported prescribing the same steroid regimen as in FECD or PBK (Fig. 4A), but with increased drop frequency (Fig. 4B). There was a tendency toward more prolonged topical steroid treatment for this patient cohort, with fewer clinicians stopping treatment at 12 months (n = 10; 13%). Most surgeons continue prescribing prednisolone 1% eye drops beyond the 12th postoperative month (n = 30, 38% during months 12–24; n = 25, 32% after 24 months). After 24 months, only 17 (22%) participants had ceased steroid eye drop use, whereas 61 (78%) surgeons continued topical steroids. Among these, 54 respondents with OD administration regime, four respondents on alternate days, two respondents twice a day, and one respondent four times a day. Most surgeons continue indefinitely, whereas three surgeons for 5 years, two surgeons for 3 years, and one surgeon OD through year 3, then on alternate days through year 4, and twice weekly during year 5.

### Post-DMEK Follow-Up

Forty-three surgeons (55%) offered indefinite follow-up, usually on an annual basis, after DMEK ±cataract surgery. Conversely, 45% of the surgeons discharged their patients after follow-up periods varying from less than 1 year to 5 years postoperatively: 8% of participants discharged their patients less than 12 months after the procedure, with another 8% doing so at 12 months. At 2 years postoperatively, 10% of surgeons discharged their patients, whereas 4% did so after 3 and 5 years, respectively. Another 15% of respondents varied their discharge decision depending on patient and graft characteristics.

## DISCUSSION

This international survey canvassed the practice patterns of 78 corneal transplant surgeons from 15 countries, with the United States being the most common country of practice (42%). Our data demonstrate variability in surgeons’ practice, building upon the results of similar surveys exploring steroid usage patterns after EK.^10,11^

Among our respondents, prednisolone 1% was the most common agent used in the first 12 months after routine DMEK or phaco-DMEK, irrespective of the indication. It remained the preferred steroid for up to 24 months postoperatively in patients with PBK and continued to be after 24 months in repeat grafts. These findings align with the existing literature, in which prednisolone 1% was identified as the most frequently prescribed steroid across all keratoplasty techniques and postsurgery intervals.^10–12^ Similarly, we identified a decrease in prednisolone 1% and dexamethasone 0.1% use after 6 months postoperatively, with a concurrent increase in use of lower potency steroids, including FML 0.1% and loteprednol 0.5%. FML was the preferred steroid 12 months after DMEK in FECD and at 24 months after DMEK for PBK. These results may be explained by the relatively low rejection risk of routine DMEK surgery,^13^ which can be safely controlled with lower-potency steroids that carry a smaller risk of adverse effects such as steroid-induced ocular hypertension or glaucoma.^6,9,14–18^ Conversely, in higher risk cases such as repeated DMEKs, prednisolone 1% was selected most often (32%) at all postsurgical intervals, even 24 months after surgery. Interestingly, in 2021, a US-based survey identified difluprednate 0.05% as the second most prescribed steroid during the first 3 months after any form of keratoplasty.^10^ By contrast, our results suggest that its use may have diminished in the interim. Only one respondent reported its use after DMEK surgery despite its high potency and relatively favorable side effect profile of difluprednate.^19^ In contrast to the survey by Boychev et al,^10^ our study included data from non–US-based ophthalmologists, which may partially explain this observed difference in practice pattern. However, despite its global nature, 42.3% of our respondent were based in North America, making it unlikely that geographic differences fully account for this observed change in practice. Future studies are needed to monitor this trend and explore the underlying reasons.

Across all 3 indications for graft surgery explored in this survey, the most popular postoperative regimen in the first 3 months was QID, regardless of steroid employed, as noted in previous research.^1^ Approximately one third of respondents initially prescribe an intensified steroid regime in the first postoperative month (≥6 × day), especially in repeated grafts. Evidence supports this approach, which may reduce incidence of postoperative cystoid macular edema and, possibly, reduces the risk of rejection.^20–22^ From the fourth month to the end of the first postoperative year, topical corticosteroids were tapered gradually in line with the published literature.^7,9,23^ In general, there was a tendency toward more frequent and prolonged corticosteroid regimen for DMEKs in PBK and after a failed DMEK compared with FECD. Beyond 12 months, OD administration was the most popular among respondents, in all 3 clinical scenarios. This finding mirrors results from previous investigations.^10,12^

The long-term use of low-dose topical steroids to reduce the risk of corneal graft rejection remains poorly studied. Studies have identified a graft rejection rate of 4% over 10 years in low-risk DMEKs, rising to 21% in high-risk grafts such as those done for previous graft failure.^24^ Only one prospective parallel-group study investigated differences in rejection rates in patients who discontinued corticosteroids 1 year after DMEK.^25^ Although the authors only reported one incidence of graft failure, rejection episodes occurred in 6% of grafts in the second year, if topical steroids were stopped after 12 months. By contrast, continuation of even low-potency corticosteroids beyond the first year appeared protective with no rejection episodes reported in that cohort.^25^ Similar evidence exists for penetrating keratoplasties.^14,26,27^ The risk of rejection needs to be balanced against potential adverse events and financial implications of indefinite postoperative corticosteroid treatment for patients and health services.^11^ Our study is the first to explore real-life practice patterns on the duration of corticosteroid therapy after DMEK. Interestingly, almost half of the respondents (44%) discontinued corticosteroids 2 years after low-risk DMEK for FECD, and 36% did so for the PBK cohort. For each of the surveyed graft indications, over half of the surgeons continued topical steroids indefinitely and did not discharge patients after DMEK. These findings highlight variations in clinical practice that require further investigation. If graft rejection episodes do not increase with cessation of topical corticosteroids after 5 years compared with indefinite treatment, patients may be safely discharged from long-term follow-up. Given the ever-growing cohort of patients with DMEK, safe transfer of care from the hospital eye service to the community can increase clinic capacity, reduce costs for patients and hospitals, and reduce the carbon footprint associated with patient travel to hospitals.

It is important to acknowledge that our study has several limitations. This study did not explore any differences in graft rejection or failure rates based on frequency and duration of postoperative corticosteroid therapies. We hypothesize that the incidence graft rejection was likely broadly in line with the published literature, as otherwise the surgeons would likely alter their practice. However, a prospective study is required to answer this question. The cross-sectional design of this study builds on previous surveys by providing a snapshot of current practice patterns and some insights into changes over time.^10–12^ Despite sampling a relatively large international cohort, respondents from high-income countries—particularly the United States (42%)—were overrepresented, potentially limiting the generalizability of the findings. Postoperative steroid usage patterns of surgeons practicing in well-resourced settings may not accurately reflect those of their counterparts in lower-income or low-resource regions, where the availability and affordability of medications can differ substantially. Furthermore, treatment strategies may be influenced by local or regional clinical guidelines and regulatory approval status of specific corticosteroids. However, because of the limited number of respondents from some regions, a meaningful geographic breakdown (eg, US, UK, EU, Asia) was not feasible. Future studies should aim to include a more representative sample from diverse practice and income settings to enhance the validity and global applicability of the results.

In conclusion, this survey provides the most recent insight into preferred corticosteroid regimen for the postoperative management of DMEK surgery in FECD, PBK, and repeat endothelial corneal transplants. Prednisolone 1% remained the commonest regimen for the first 3 postoperative months, with low-potency steroids, such as FML, typically used thereafter. We identified significant variability in surgeons’ practice, most notably regarding the duration of topical corticosteroid use and long-term patient follow-up after DMEK. In general, this heterogeneity in current practice patterns calls for an international consensus on the optimal postoperative corticosteroid therapy after endothelial keratoplasty.

## Supplementary Material
