# Supplementary material for: Survey of Topical Steroid Usage Patterns After Descemet Membrane Endothelial Keratoplasty
Source: Cornea. 2025 Aug 5;45(8):968–73. doi: 10.1097/ICO.0000000000003953 (PMC13317915; doi:10.1097/ICO.0000000000003953)
Supplement: Supplementary file 1 [file cornea-45-0968-s001.pdf]

Default Question Block

In which city and country do you practise?

[ ]

Approximately how many DMEKs do you perform per year?

[ ]

Which steroid eye drops do you use after routine DMEK or phaco/DMEK for Fuchs endothelial dystrophy?

	Steroid Medication (name and strength)	Frequency (per day)	Stop Medication
First month			
Months 1-3			
Months 4-6			
Months 7-12			
Months 12-24			
After 24 months			

If you continue steroids after 24 months, for how long do you continue?

[ ]

Which steroid eye drops do you use after routine DMEK for pseudophakic

bullous keratopathy?

	Steroid Medication (name and strength)	Frequency (per day)	Stop Medication
First month			
Months 1-3			
Months 4-6			
Months 7-12			
Months 12-24			
After 24 months			

If you continue steroids after 24 months, for how long do you continue?

Which steroid eye drops do you use for DMEK following previous failed DMEK (repeat graft)?

	Steroid Medication (name and strength)	Frequency (per day)	Stop Medication
First month			
Months 1-3			
Months 4-6			
Months 7-12			
Months 12-24			
After 24 months			

If you continue steroids after 24 months, for how long do you continue?

____

Do you usually discharge patients from your clinic/service after a routine DMEK or phaco/DMEK procedure?

- ○ __________ Yes (please specify after how many months or years)
- ○ No (indefinite follow-up)

Please include any other information on your steroid tapering regime or consideration following DMEK that you would like to share.

____

Powered by Qualtrics
